# Predicting the splash of a droplet impinging on solid substrates

**DOI:** 10.1038/s41598-022-08852-3

**Published:** 2022-03-24

**Authors:** Yukihiro Yonemoto, Kanta Tashiro, Kazuki Shimizu, Tomoaki Kunugi

**Affiliations:** 1 Division of Industrial Fundamentals, Faculty of Advanced Science and Technology, Kumamoto University, 2-39-1, Kurokami, Chuo-ku, Kumamoto-shi, Kumamoto 860-8555 Japan; 2 Department of Mechanical and Mathematical Engineering, Kumamoto University, 2-39-1, Kurokami, Chuo-ku, Kumamoto-shi, Kumamoto 860-8555 Japan; 3 College of Energy Engineering, Zhejiang University, 38 Zheda Road, Hangzhou, Zhejiang Province 310027 People’s Republic of China

**Keywords:** Fluid dynamics, Surfaces, interfaces and thin films

## Abstract

The impingement behaviours of droplets towards solid substrates depend on the liquid properties, impingement velocity and solid surface conditions, such as wettability and roughness. However, the prediction regarding whether the droplet splashes after the impingement, is still an open question. Here we show that the splashing can be predicted by the pressure balance of the liquid film appearing beneath the impingement droplet coupled with the modified energy balance equation. Hydrodynamic and hydrostatic pressures are the driving forces for the droplet’s radial spreading, while the capillary pressure at the rim edge and viscous stress oppose the driving forces. Thus, splashing occurs when the driving forces overcome the opposing forces. Moreover, the splashing condition is affected by various surface factors, such as wettability and surface roughness. Our work would pave the way to understand the basic physics for rim or liquid film fragmentation and enabling advances in important for engineering field such as printing, sprays for cooling and pesticide.

## Introduction

Droplet behaviour are of major significance in various fields, particularly industrial and agricultural fields including various processes, such as coating, ink-jet printing, spray cooling and pesticides for plants^1–3^. From the environmental perspective, the rainfall hitting the soil is one of key factors for aerosol generation^4^. In addition, the droplet behaviours against solid substrates, such as rebound/splash and deposition^5^ are concerning for the field of epidemiology as the splash-back in a public lavatory and the dispersion and transmission of potential virus vectors causing infectious respiratory diseases, e.g. the interaction between mask and saliva droplets^6,7^. Thus, deeper understanding of droplet behaviours is crucial for the development and improvement of technologies in the above-mentioned fields. However, there are several behaviours that are unclear on droplet impinging on solid substrates. In particular, whether the droplet splashes or not continues to be an open question, although numerous researchers have investigated this topic^8–15^.

Almohammadi and Amirfazli^16^ investigated the droplet behaviour for different liquids with a wide range of viscosities and surface tensions on smooth, hydrophilic and hydrophobic substrates. In the study, they proposed an empirical model to predict whether a droplet splashed or not after impinging on solid substrates. Riboux and Gordillo^17^ performed the experiment for droplet splashing with eight types of liquids and proposed a model to determine the critical velocity of a splashing on a smooth and dry solid surface. In the model, they focused on the local lamellar behaviour of the droplet. The main concept of this model considered the balance between the vertical lift force acting on the edge of the lamella because of the lubrication and suction forces imparted by gas and the retraction force by a capillary. In the model, the angle of the lifted rim edge was the key parameter. The ratio of the two forces was called the splashing parameter, and the splash occurred if this parameter was greater than 0.14. Quetzeri-Santiago et al.^18,19^ performed the experimental observation for the splashing behaviours of water and ethanol droplets on smooth and rough solid substrates. They highlighted that the splashing parameter qualitatively has a linear relationship with *R*_pk_/*R*_sm_ (the ratio of the peak to peak roughness *R*_pk_ and the mean width of the surface features *R*_sm_) and the maximum dynamic contact angle θ_max_, implying that the lamellar separation from the solid substrate depends on the wettability. Recently, the experimental and analytical studies were performed for millimetric droplets of water and ethanol impinging on the substrates finished by sandpapers with different roughness values^20^. In the research, the rim behaviour of the liquid film was elaborated. Based on the local liquid film behaviour, the models for predicting the critical Weber number (We = *ρu*^2^*r*_0_/σ) where *ρ* is the liquid density, *u* is droplet impinging velocity, *r*_0_ is initial droplet radius and σ is surface tension for splashing are categorised into different cases depending on the magnitude of We_ε_: We_ε_ is the Weber number evaluated by the grit size (ε) of the sandpaper as *ρu*^2^ε/σ. Especially, the critical We is simply correlated to (*r*_0_cos θ_0_/ε)^3/5^ when We_ε_ $\ge$ 1, where θ_0_ is static contact angle when the liquid wets the substrate. Although, investigating the local liquid film behaviour has helped increase our understanding of the mechanism behind the splashing behaviour of the droplets, the researchers have still not reached a consensus in their findings^21–25^.

Recently, Yonemoto and Kunugi^26^ proposed an analytical model for the prediction of the maximum spreading contact-area diameter of the droplets based on the energy balance approach. The model was in good agreement with the experimental data covering various liquid viscosities and surface tensions^27^. This model suggested that the droplet ideally spreads over smooth solid surfaces without splashing. However, for practicality, the understanding of the droplet wetting behaviour on rough solid surfaces, including deposition and splashing, is very important^18–20,26–28^. The spreading and retracting processes of the droplet mainly depend on its inertia and wettability, complicating the evaluation of the maximum spreading area^29^. For both smooth and rough surfaces, droplet splashing occurs during its spreading. Therefore, in the present study, we have modified the energy balance equation for both smooth and rough solid surfaces and developed a novel model for predicting the splashing condition of a droplet. This model combines the pressure balance of the liquid film with a simplified circumferential-instability model. Finally, the splashing criterion is obtained by solving the modified energy balance equation combined with the newly developed pressure balance relation. The developed model can predict the splashing condition on both smooth and rough solid substrates. In addition, the modified energy balance equation can also predict the spreading contact-area diameter in the deposition region on both the substrates.

## Methods

Water–ethanol binary mixtures were used in our study. The mass concentration was varied from 0 to 99.4 wt.% ethanol (pure ethanol; Nacalai Tesque, Inc., 99.4 wt.%). Here, the viscosity of the water–ethanol binary mixtures exhibits the maximum value at around 40 wt.%. Therefore, we chose the weight percentage of 6 cases to investigate the effect of the surface tension on the splashing behaviour under similar liquid viscosity µ_l_. Concretely speaking, the ultrapure water (Wako Pure Chemical Industries, Ltd., σ_lg_ = 0.0719 N m^−1^ and µ_l_ = 1.00 m Pa s), four water–ethanol binary mixtures (σ_lg_ = 0.0563 N m^−1^ and µ_l_ = 1.25 m Pa s (5 wt.%), 0.0384 N m^−1^ and µ_l_ = 2.18 m Pa s (20 wt.%), 0.0301 N m^−1^ and µ_l_ = 2.91 m Pa s (40 wt.%), 0.0256 N m^−1^ and µ_l_ = 2.37 m Pa s (70 wt.%)) and pure ethanol (0.0211 N m^−1^ and µ_l_ = 1.20 m Pa s (99.4 wt.%)) were used in the experiments. The surface tensions of the liquids were measured using the DM300 (Kyowa Interface Science Co., Ltd.). The literature data were used for the liquid viscosity^30^.

Polycarbonate (PC) was used as the solid material. To evaluate the effect of the surface roughness on the droplet splashing behaviour, the solid material was polished with a grinder-polisher (MetaServ^TM^ 250 Grinder-Polisher, Buehler Ltd., Lake Bluff, IL, USA). The surface was prepared by using three types of polishing sheets, 408-400AU (Grit #400), 408-240AU (Grit #240) and 408-120AU (Grit #120), Sankei Co. Ltd., JAPAN. Silicone rubber (SR) substrate was also used as an additional hydrophobic substrate with negligible adsorption. The surface morphological conditions were measured using a laser scanning microscope (LEXT OLS4100, Olympus Co. Ltd., JAPAN). The arithmetical mean-roughness value (*R*_a_) was measured for each substrate as 0.03 µm for the bare plate, 0.33 µm for #400 plate, 0.99 µm for #240 plate and 1.25 µm for #120 plate. *R*_a_ for SR was 0.02 µm. Prior to the experiment, the solid materials were rinsed with ethanol and purified water for the SR substrate and the purified water for the PC substrates. Then, the materials were dried with an air blower.

The droplets were gently released without any initial velocity using a micro-syringe from different heights (5–2400 mm). The droplet volume ranged from 3.4 to 6.6 µL. The errors in the droplet volumes of the droplets were within 3% in the present study. In addition, the difference between the measured vertical and horizontal initial droplet diameters were within 10%^31^, indicating a good repeatability of the experiment. A high-speed video camera (HX-5, NAC image technology, Ltd., Japan) with a microscope (Leica Microsystems, Welzlar, Germany) or a micro-lens (Nikon AF-S VR Micro-Nikkor 105 mm f/2.8G IF-ED) was used to capture the impingement behaviour with the frame rate of 20,000 fps. Each condition was repeated three times. The critical splashing velocity of a droplet was determined by observing the secondary droplet falling from the same height thrice. The validity of the present experiment was confirmed by comparing with the existing splashing models (Section S5 in the supplementary information). Notably, the identification of the splashing type, prompt or corona, is out of scope for the present study^32–35^. The temperature and relative humidity were maintained within 20.0–25.0 ℃ and 50.0–55.0%, respectively.

## Experimental results

### Spreading of droplets on smooth and rough solid surfaces

**Figure 1:**
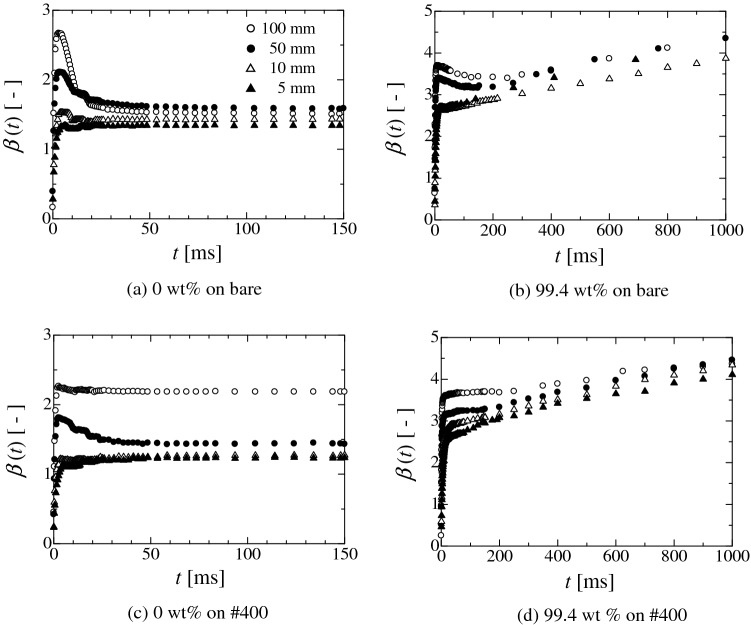
Time evolution of normalised contact-area diameter *β*(*t*) (= *d*_cont_(*t*)/*d*_0_: *d*_cont_ the contact-area diameter, *d*_0_ the initial droplet diameter). The symbols of solid triangle, open triangle, open circle and solid circle represent the release heights of the droplet from 5, 10, 50 and 100 mm, respectively. The droplet is released from each height with no initial velocity.

**Figure 2:**
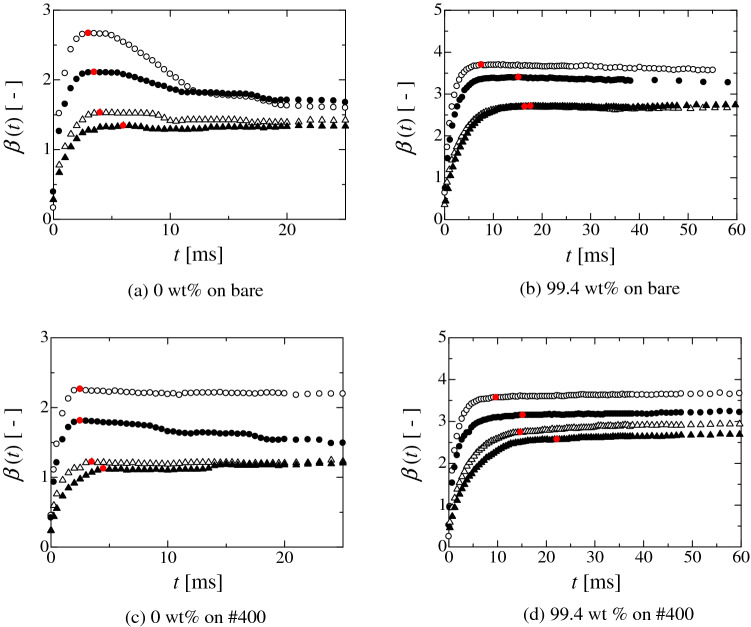
Time evolution of normalised contact-area diameter *β*(*t*) just after impingement (Magnified image for the earlier stage in Fig. S1). Red solid circle represents the first stationary point where d*β*(*t*)/d*t* $\approx$ 0. the solid triangle, open triangle, open circle and solid circle represent the release heights of the droplet from 5, 10, 50, and 100 mm, respectively. The droplet is released from each height with no initial velocity.

In the droplet impingement process, the droplet shape exhibits a damped vibration process as the time advances. The droplet shape change also affects the behaviour of the contact-area diameter *d*_cont_(*t*), here, *t* is the time. For weak hydrophilicity, the change in the contact-area diameter post-impingement reaches 0, which corresponds to the stationary point represented by d*d*_cont_(*t*_stp_)/d*t* $\approx$ 0, here, *t*_stp_ is the time at the stationary point. Hereafter, the contact-area diameter *d*_cont_(*t*_stp_) or radius *r*_cont_(*t*_stp_) at the stationary point is represented by *d*_stp_ or *r*_stp_. The size of the contact-area diameter at the second stationary point is usually smaller than the first one. However, for strong hydrophilicity, because of the intermolecular interaction or the effect of surface roughness on wettability, the behaviour of the contact-area diameter becomes complicated. Figure 1 shows the time evolution of the normalised contact-area diameter *β*(t) (= *d*_cont_(*t*)/*d*_0_: *d*_0_ is the initial droplet diameter) for (a) 0 wt.% and (b) 99.4 wt.% on the bare plate and (c) 0 wt.% and (d) 99.4 wt.% on #400 plate. The droplet is released from different heights of 5, 10, 50 and 100 mm. Figure 2 depicts the earlier stage in Fig. 1 and shows the time evolution of *β*(*t*) post droplet impingement. In Fig. 2, the red points represent when the change in *β*(*t*) approximately becomes 0 (i.e. the first stationary point of d*β*(*t*_stp_)/d*t* $\approx$ 0). Here, the *β*(*t*_stp_) is the normalised contact-area diameter at the stationary point. Hereafter, *β*(*t*_stp_) is represented by *β*_stp_. Evidently, for (a), the contact-area diameter reaches the stationary point just after the droplet impingement for each release height. Subsequently, *β*(*t*) becomes small (as in the retraction process) and reaches a constant value. For each release height, the stationary point assumes the maximum value because *β*(*t*) after the first stationary point does not exceed the value of *β*(*t*_stp_). In the case of (b), *β*(*t*) reaches the stationary point just after the impingement and exhibits the retraction process for each release height (Fig. 2). However, after retracting, *β*(*t*) gradually increases again and becomes larger than the value at the first stationary point. Basically, the static wetting condition of 99.4 wt.% ethanol exhibits complete wetting of the bare plate^36^. This means that the droplet continues to spread over the solid surface during the droplet impingement process. Therefore, the adsorption effect on the contact line motion becomes prominent after the kinetic energy is almost consumed. This result indicates that there are two processes for the droplet spreading^29^. One involves inertia wherein the spreading is promoted by the fluid motion. The other one is wettability dominated process in which the spreading is promoted by the molecular scale interaction. Moreover, the surface morphology also affects the spreading, as shown in Figs. 1c, d and 2c, d. In these cases, the droplet behaviour becomes more complicated than that in the bare substrate because the surface roughness induces the pinning of the contact line, making it hard to recede. In the (c) and (d) cases, the retraction of the contact line after the first stationary point becomes small. Therefore, *β*(*t*) after the first stationary point exhibits various behaviours depending on the release height, wettability and surface roughness. In other words, it depends on the behaviour of the contact-area diameter after the first stationary point whether the stationary point becomes the global maximum, local maximum, or inflection point. If the stationary point contains the largest value through the whole droplet impingement process, the spreading contact-area diameter becomes the maximum spreading contact-area diameter. At least, the droplet spreading behaviour until reaching the first stationary point is the inertia dominated process. Therefore, there is no physical contradiction to the prediction of the spreading contact-area diameter of the droplet at the first stationary point using the energy balance approach (Section S4).

### Splashing of droplets on solid substrates

**Figure 3:**
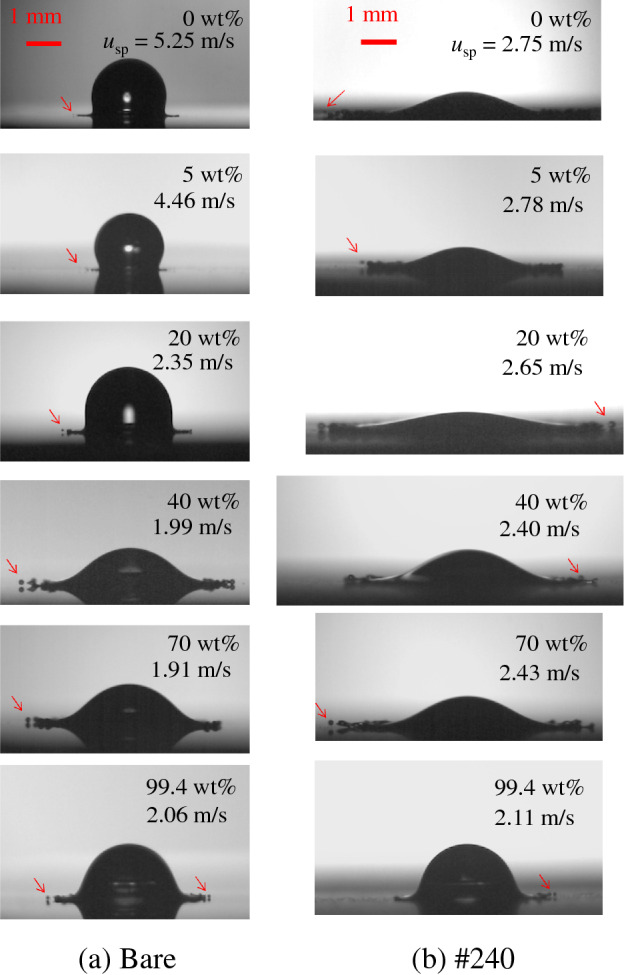
Splashing images of water–ethanol binary-mixture droplets on (a) bare (smooth solid surface) and (b) #240 PC substrates. The red arrows indicate the ejected secondary droplets. The droplet volumes for each liquid are 4.5 µL for 0 wt.%, 3.4 µL for 5 wt.%, 6.6 µL for 20 wt.%, 5.3 µL for 40 wt.%, 5.3 µL for 70 wt.% and 4.9 µL for 99.4 wt.%.

Figure 3 shows the splashing images of water–ethanol binary-mixture droplets on the bare (smooth solid surface) and #240 PC substrates. For bare substrate, the splashing velocity of the water droplet is larger than that of other liquids. Conversely, for #240 PC substrate, the differences in the splashing velocities are small in terms of the liquid concentration. For low concentrations (0 wt.% and 5 wt.%), the splashing velocities on the bare substrate are larger than those on other roughened solid substrates. Similar tendency has been observed for water in another study^20^. The impingement velocities for PC substrates where the splashing occurs are listed in Table 1. In the table, the impingement velocities for SR substrate are also shown.

**Table 1:** Impingement velocity *u*_c_ [m/s] of each droplet where the splashing occurs on each solid substrate.

Ethanol concentration *w* [wt%] and initial droplet volume *V*_0_ [µL]	PC				SR
	Bare	#400	#240	#120	Bare
0 wt.% (4.5 ± 0.0 (PC), 4.7 ± 0.0 (SR))	5.28 ± 0.16	2.82 ± 0.06	2.75 ± 0.16	2.30 ± 0.05	5.07 ± 0.17
5 wt.% (3.4 ± 0.0)	4.46 ± 0.09	2.82 ± 0.13	2.77 ± 0.02	2.47 ± 0.08	−
20 wt.% (6.6 ± 0.2 (PC), 7.2 ± 0.3 (SR))	2.35 ± 0.07	2.62 ± 0.11	2.64 ± 0.19	2.18 ± 0.12	2.54 ± 0.11
40 wt.% (5.3 ± 0.2 (PC), 5.5 ± 0.3 (SR))	1.99 ± 0.08	2.28 ± 0.10	2.40 ± 0.14	2.42 ± 0.03	2.01 ± 0.05
70 wt.% (5.3 ± 0.1)	1.92 ± 0.03	2.22 ± 0.07	2.43 ± 0.04	2.34 ± 0.01	−
99.4 wt.% (4.9 ± 0.1 (PC), 4.3 ± 0.0 (SR))	2.06 ± 0.02	2.13 ± 0.03	2.11 ± 0.07	2.13 ± 0.06	2.32 ± 0.08

## The model for splashing

### Pressure balance of the liquid film

**Figure 4:**
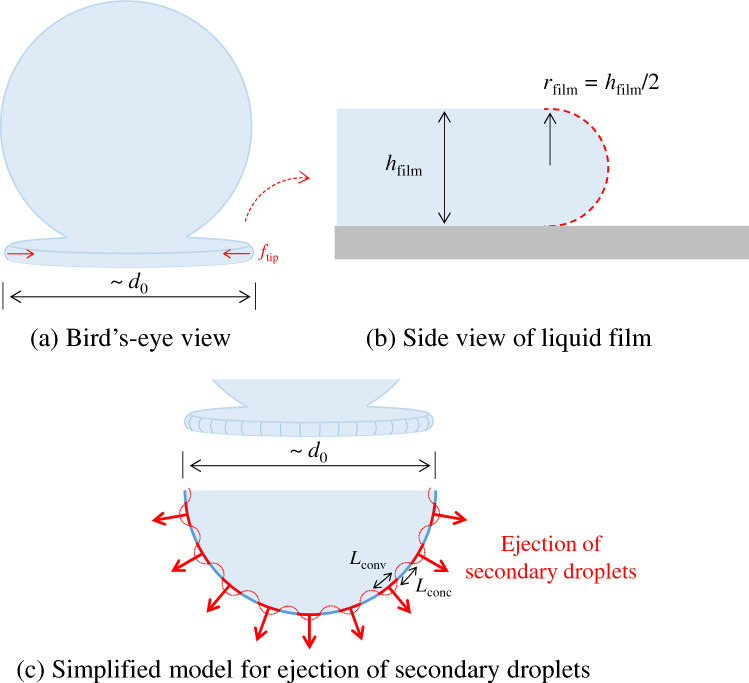
Schematic of the simplified model for liquid film behaviour in droplet impingement. (a) The droplet and liquid film at a moment in the impingement. The surface tension exerted on the tip of the liquid film is expressed as *f*_tip_. (b) Side view of the tip of the liquid film with the thickness of *h*_film_, and the tip shape is characterised by a part of circle whose radius is *r*_film_ (= *h*_film_ / 2). (c) Circumferential instability of the liquid film is simplified, and the diameter of the disc-like liquid film is characterised by *d*_0_. *L*_conv_ and *L*_conc_ are the arc lengths of the concave and convex parts projected on the circumference. The secondary droplet is assumed to eject from every convex position along the circumference (red bold line).

The typical image for the droplet impingement process reflects that the liquid film spreads over the solid surface just after the droplet impingement, and the tip of the liquid film reaches the first stationary condition d*d*_cont_(*t*_stp_)/d*t *$\approx$ 0, where the contact-area diameter *d*_cont_(*t*) achieves the maximum value and the droplet exhibits retraction. Finally, the droplet reaches equilibrium through the damped vibration process. The droplet splashing occurs in a series of the impingement process if the kinetic energy is sufficient to eject the secondary droplets from the tip of the liquid film. As shown in Fig. 4a, the existence of the liquid film when the droplet impinges on the solid substrate is very important for the discussion of the splashing behaviour^17^. Especially, it can be assumed that the splashing occurs when the secondary droplets are generated from the tip of the disc-like liquid film. The pressure balance is considered for this concept^37^.

At a moment in the droplet impingement, the liquid starts spreading in the radial direction, and the hydrodynamic pressure, *P*_hyd_, driving the liquid film in the radial direction is expressed using the initial radial velocity *u*_H_^0^ as follows:

1 $$P_{\text{hyd}} = \frac{1}{2}\rho_{\text{l}} \left( {u_{\text{H}}^{0} } \right)^{2} .$$

In Eq. (1), *ρ*_l_ is the liquid density and the initial radial velocity *u*_H_^0^ is assumed to be 3*u*/8, where *u* is the impinging velocity perpendicular to the solid substrate. *u*_H_^0^ (= 3*u*/8) can be estimated by considering mass conservation where a cylindrically shaped droplet of diameter *d*_0_ is assumed for the sake of convenience^26^. Then, the hydrostatic pressure *P*_stat_, another driving force is expressed as follows:

2 $$P_{\text{stat}} = \rho_{\text{l}} gd_{0} .$$

In this equation, *d*_0_ represents the initial droplet diameter and *g* is the gravity. Then, if a thin disc-like liquid film protrudes in the radial direction at a moment in the impingement, the surface tension exerted on the tip of the liquid film (*f*_tip_), as shown in Fig. 4a, b, is expressed as *f*_tip_ = $(2\pi r_{\text{film}} \sigma_{\lg } /2) + (2\pi r_{0} \sigma_{\lg } /2)$ characterized by *r*_film_ and *r*_0_ (= *d*_0_/2), where σ_lg_ is the surface tension between the liquid and gas. Then, a simplified model of the circumferential instability to the liquid film is applied in the study, as shown in Fig. 4c, where the concave (*L*_conc_) and convex (*L*_conv_) arc lengths are approximated the same: *L*_conc_ $\approx$ *L*_conv_ (Section S1 in the supplementary information). A secondary droplet is assumed to eject from every convex position along the circumference based on the simplified circumferential-instability model. From this assumption, the effective area *A*_eff_ on the tip of the liquid film where the surface tension exerts can be modelled as *h*_film_ $\times$ (2*πr*_0_ $\times$ 1/2). Finally, the Laplace pressure *P*_Lap_ exerted on the tip of the liquid film can be derived from *f*_tip_/*A*_eff_ as follows:

3 $$P_{\text{Lap}} \approx \left( {\frac{1}{{d_{0} }} + \frac{1}{{h_{\text{film}} }}} \right)\sigma_{\lg } .$$

In addition, the viscous stress *P*_vis_ is expressed by

4 $$P_{\text{vis}} \approx \mu_{\text{l}} \frac{{u_{\text{H}}^{0} }}{{h_{\text{eff}} }}.$$

In this relation, the effective height *h*_eff_ is evaluated by *h*_film_/3 based on the wall jet flow and parallel plate flow^26^. From Eqs. (1)–(4), the secondary droplet ejected from the tip of the liquid film is generated when the following relation holds:

5 $$P_{\text{hyd}} + P_{\text{stat}} \ge P_{\text{Lap}} + P_{\text{vis}} .$$

The boundary condition whether the droplet splashed or not is *P*_hyd_ + *P*_stat_ = *P*_Lap_ + *P*_vis_.

A precise prediction of the size of the secondary droplet ejected from the liquid film is very difficult. Therefore, in the present study, the secondary droplet size is characterised by the droplet size of *h*_stp_ evaluated by postulating a disc-shaped droplet as *V*_0_/π*r*_stp_^2^, where *r*_stp_ is the spreading contact-area radius at the first stationary condition, *V*_0_ is the volume of droplet and *h*_stp_ is the droplet height at *r*_stp_^26^. Here, the first stationary condition represents the situation in which the droplet spreads just after the impingement and the change in the contact-area diameter *d*_cont_(*t*) approximately becomes zero (d*d*_cont_(*t*_stp_)/d*t* $\approx$ 0). The droplet spreading behaviour until reaching the first stationary point is the inertia dominated process (“Spreading of droplets on smooth and rough solid surfaces” section). Thus, if the contact-area diameter in the first stationary condition holds the largest value throughout the droplet impingement process, the spreading contact-area diameter is called the maximum spreading contact-area diameter *d*_m_. It depends on the behaviour of *d*_cont_(*t*) after the first stationary condition whether the stationary point becomes the global maximum, local maximum or inflection point. Furthermore, the secondary droplet size is affected by the wettability because the spontaneous capillary-driven spreading will occur when the droplet contacts the solid substrate^38,39^. Even in the case of an impact of a solid body in a liquid bath, the wettability affects the splashing behaviour of the liquid^40^. Therefore, based on the above concept, the thickness of the liquid film *h*_film_ can be modelled as follows (Section S2 in supplementary information):

6 $$h_{\text{film}} = \left( {1 + f(\overline{\uptheta }^{i} )\cos \overline{\uptheta }^{i} } \right)^{1/3} \frac{2}{3}\frac{{d_{0}^{3} }}{{d_{\text{stp}}^{2} }}.$$

In this equation, *f* ($\overline{\uptheta }^{i}$) = (1 + cos $\overline{\uptheta }^{i}$)/2 where $\overline{\uptheta }^{i}$ (*i* = bare or rough) is the average contact angle of $\uptheta_{\text{st}}^{i}$ and $\uptheta_{\text{stp}}^{i}$ defined in Eq. (A12): $\uptheta_{\text{st}}^{i}$ and $\uptheta_{\text{stp}}^{i}$ represent the static contact angle and the contact angle at which the contact-area radius reaches the stationary condition on each solid substrate. Notably, the present study does not focus on the quantitative evaluation of the secondary droplets. For the detailed quantitative evaluation of the secondary droplet, the fluid flow and vortex behaviour of the droplet impinging on the solid substrate must be investigated because the Görtler vortices^41,42^ may be related to the instability in the liquid film-tip behaviour.

### Effect of surface roughness on spreading

Wettability of droplet on the solid substrate is affected by the surface morphology and the interaction between the solid and liquid molecules. The intermolecular interaction is an essence of the physics of wettability. Therefore, the adsorption of liquid molecules on a solid substrate is an important phenomenon^43^. The difference in the strength of intermolecular interaction would be indirectly exhibited by an indicator for wettability, such as contact angle. For example, the adsorption of liquid molecules on a hydrophobic substrate^44^ is not as significant as that on a hydrophilic one^36^. This is inevitable for the energy balance concept used for the prediction of the spreading factor. Thus, in the present study, the conventional energy balance equation is also modified based on the same concept.

#### Morphological effect on radial spreading velocity

As the surface roughness increases, the effect of the solid surface on the fluid motion would also increase, inducing changes in the viscous force and dissipation. This type of morphological effect must be considered in the pressure balance model and conventional energy balance equation^26^. The evaluation of the roughness effect strictly on the fluid motion was difficult although the relationship between the radial spreading velocity and surface conditions, such as wettability and roughness, was highlighted^20^. Therefore, in the present study, the morphological effect is evaluated by considering the relationship between the work of adhesion for solid–liquid and the hydrodynamic force radially pushing the droplet. Therefore, hydrodynamic force comparable to the work of adhesion is at least needed to push the liquid forward in a radial direction. For example, for a bare substrate, the relation among the initial spread diameter *d*_bare_, the initial radial velocity *u*_H_^0,bare^ and the contact angle $\overline{\uptheta }_{{}}^{\text{bare}}$ can be evaluated as follows:

7 $$\frac{1}{2}\rho_{\text{l}} \left( {u_{\text{H}}^{0,\text{bare}} } \right)^{2} \pi d_{\text{bare}}^{{}} d_{0} \sim \pi d_{\text{bare}}^{{}} \sigma_{\lg } (1 + \cos \overline{\uptheta }^{\text{bare}} ).$$

For a rough substrate, the relation among the initial spread diameter of *d*_rough_, initial radial velocity of *u*_H_^0,rough^ and contact angle of $\overline{\uptheta }_{{}}^{\text{rough}}$ can be considered as follows:

8 $$\frac{1}{2}\rho_{\text{l}} \left( {u_{\text{H}}^{0,\text{rough}} } \right)^{2} \pi d_{\text{rough}}^{{}} d_{0} \sim \pi d_{\text{rough}}^{{}} \sigma_{\lg } (1 + \cos \overline{\uptheta }^{\text{rough}} ).$$

By taking the ratio of Eqs. (7)–(8), the following relation is deduced:

9 $$\left( {\frac{{u_{\text{H}}^{0,\text{bare}} }}{{u_{\text{H}}^{0,\text{rough}} }}} \right)^{2} \sim \frac{{1 + \cos \overline{\uptheta }^{\text{bare}} }}{{1 + \cos \overline{\uptheta }^{\text{rough}} }}.$$

Equation (9) implicitly indicates the morphological effect on the fluid motion. Therefore, if *u*_H_^0,bare^/*u*_H_^0,rough^ is defined as *k*, it is related to the radial spreading velocity in Eqs. (1) and (4). Thus, from Eqs. (1)–(5), the following equation can be derived:

10 $$\left( {\frac{3}{2} + \frac{27}{{16}}k\frac{\text{We}}{{\text{Re}}}} \right)\beta_{\text{stp}}^{2} \le \left( {1 + f(\overline{\uptheta }^{i} )\cos \overline{\uptheta }^{i} } \right)^{1/3} \left( {\frac{{9k^{2} }}{128}\text{We} - 1 + \frac{{\rho_{\text{l}} gd_{0}^{2} }}{{\sigma_{\lg } }}} \right),$$

where *β*_stp_ is the factor of the spreading contact-area diameter at the first stationary condition. In the present model, We and Re are evaluated by the initial droplet diameter *d*_0_ like as We = *ρ*_l_*u*^2^*d*_0_/σ_lg_ and Re = *ρ*_l_*ud*_0_/µ_l_, respectively. Similarly, *k* is incorporated in the viscous dissipation term in the energy balance equation as follows:

11 $$E_{\text{vis}}^{\text{rough}} = k^{2} \mu_{\text{l}} \left( {\frac{{u_{\text{H}} }}{{h_{\text{eff}} }}} \right)^{2} V_{0} t_{\text{stp}} ,$$

where µ_l_ is the liquid viscosity and *t*_stp_ is the time at which *r*_stp_ is reached. Thus, the viscous dissipation term in the conventional energy balance equation^26^ is replaced by Eq. (11).

#### Adsorption effect on spreading

In the present study, polycarbonate and water–ethanol binary-mixture liquids were used as the solid material and the test fluid, respectively. In this combination, for low-ethanol concentration, the more the surface roughness of the solid substrate, the less is the wettability. In contrast, if the ethanol concentration is high, the more the surface roughness of the solid substrate, the more is the wettability of the liquid^36^. However, even for bare substrate, more the ethanol concentration, the more is the wettability. In addition, the contact line would be easy to move in the advancing direction if the wettability exhibits hydrophilicity^45^. These phenomena indicate that the deformation of liquid surface can contribute to the contact line movement (radial spreading). Then, the additional work infiltrating into the surface morphology, such as protrusions or grooves would generate. In particular, polycarbonate has a hydrophilic tendency for water–ethanol binary-mixture liquids, making the adsorption important for the wetting behaviour. In microscopic point of view, the liquid molecules in the vicinity of the solid surface would behaves like a solid^46–48^ because the bonding energy of solid–liquid molecules will be larger than that of liquid–liquid molecules in the case of the PC substrates, although it would depend on the combination of solid and liquid in an actual situation and would be also influenced by the surface morphology. The effect of the solid–liquid adsorption on the liquid molecules motion will gradually decreases with increasing distance from the solid surface^49^. Thus, it can be assumed that the adsorption at the solid–liquid interface would be categorized into two parts. One is the strong adsorption effect on the liquid molecules in the vicinity of the solid surface. The other is the weak adsorption effect on the liquid molecules in the bulk side near the solid surface. The latter can consider as the adhesion energy characterised by the macroscopic information such as the contact angle and the surface tension. The former will be more microscopic interaction than the latter and cannot express by the macroscopic information. However, it would be particularly important in the dynamic process of the wetting behaviour such as the slip condition^50^ and the dynamic contact angle^51–55^. Therefore, the energy terms related to the adsorption (*E*_ads_) and the infiltration into the surface morphology (*E*_infil_) are added into the energy balance equation^26^.

12 $$E_{\text{kine}} + E_{\text{grav}} + E_{\text{surf}} = E_{\text{sprd}} + E_{\text{vis}}^{\text{rough}} + E_{\text{def}} + E_{\text{additional}}^{{}} ,$$

where *E*_kine_, *E*_surf_, *E*_grav_, *E*_sprd_ and *E*_def_ are the kinetic energy, initial surface energy, gravitational potential of the droplet, adhesion energy and deformation energy after the impingement, respectively, and the additional energy *E*_additional_ is considered as follows:

13 $$E_{\text{additional}} = E_{\text{ads}}^{{}} + E_{\text{infil}} .$$

Here, it is assumed that the energy can be indirectly estimated from *E*_def_ because the adsorption and the surface morphology affect the droplet surface deformation through the contact line motion. The concrete expression for *E*_ads_ is modelled as follows:

14 $$E_{\text{ads}} = g_{\text{ads}} E_{\text{def}}^{{}} .$$

In Eq. (14), the ratio of *E*_ads_ to *E*_def_, affected by the intermolecular interaction between the solid and liquid, is represented as *g*_ads_. As to *g*_ads_, Langmuir-type function is postulated using the surface tension of liquid (σ_lg_) and the critical surface tension of solid (σ_c_) based on the liquid molecule adsorption to the solid substrate as follows^36^:

15 $$g_{\text{ads}} = \frac{{A\left( {\frac{{\sigma_{\lg } }}{{\sigma_{\text{c}} }}} \right)^{6} }}{{1 + B\left( {\frac{{\sigma_{\lg } }}{{\sigma_{\text{c}} }}} \right)^{6} }} - 1,$$

where *A* and *B* are arbitrary parameters. In Eq. (14), the negative value for g_ads_ means that the reduction of energy for the surface deformation corresponds to the relative increase of other energies in the energy balance equation, which leads to the spreading of droplet. The positive value indicates that the energy is consumed in the surface deformation rather than the spreading. Here, the critical surface tensions σ_c_ for each PC substrate are 0.0247 N m^−1^ for bare, 0.0276 N m^−1^ for #400, 0.0303 N m^−1^ for #240 and 0.0317 N m^−1^ for #120, respectively^36^. The value of σ_c_ for SR substrate is 0.0196 N m^−1^^44^.

As for *E*_infil_, the following relation is assumed,

16 $$E_{{{\text{infil}}}} = g_{{{\text{infil}}}} E_{{{\text{def}}}}^{{}} .$$

In Eq. (16), *g*_infil_ is defined by the following relation as the synergy effect of the intermolecular interaction and the surface morphology on the wettability:

17 $$g_{{{\text{infil}}}} = \left[ {C\left( {\frac{{\sigma_{\lg } }}{{\sigma_{\text{c}} }}} \right) + 1} \right]D\left( {f - 1} \right),$$

In this equation, *C* and *D* are arbitrary parameters. *f* represents the relative surface-roughness area^36^. Here, the values *f* for PC substrates are 1 for bare plate, 1.12 for #400, 1.23 for #240 and 1.28 for #120, respectively. In the case of the SR substrate, the value of *f* is treated as 1. *E*_infil_ reflects the work for the infiltration of liquid into the surface morphology, and *g*_infil_ (= *E*_infil_/*E*_def_) qualitatively expresses the wetting states, such as Wenzel or Cassie state (Section S3 in supplementary information). The parameters *A*–*D* in Eqs. (15) and (17) are determined from the relationship between *β*_stp_ and We for the droplet impingement behaviour on roughened solid surfaces. Here, the values of *A*, *B*, *C* and *D* are 8.135, 7.823, − 0.3797 and 1.792, respectively.

Finally, the splashing condition is evaluated by both Eq. (10) and the modified energy balance equation Eq. (12) (= Eq. A10). However, the contact angles in the Eqs. (9) and (10) are evaluated by Eq. (A12) of the averaged values for the static contact angle *θ*_st_ and dynamic contact angle *θ*_stp_, where the spreading contact-area diameter reaches the first stationary condition.

## Results and discussion

**Figure 5:**
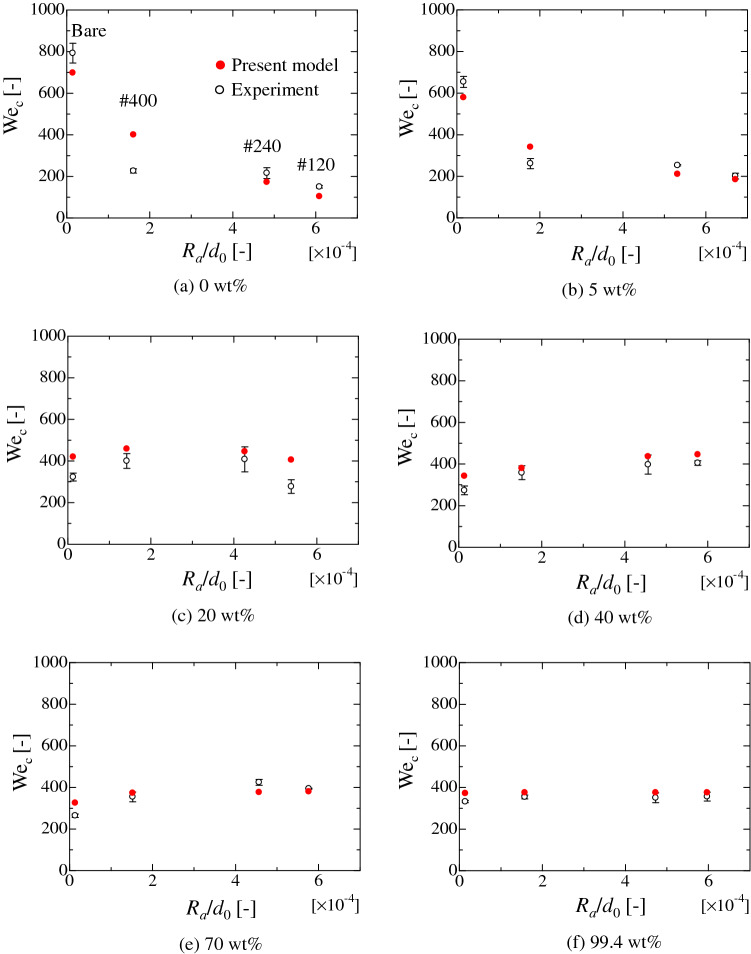
Comparison of the critical We calculated by Eqs. (A10) and (A18) with the experimental data for water–ethanol binary-mixture droplets on the solid substrates of PC. The horizontal axis reflects the arithmetical mean-roughness value *R*_a_ normalised by the initial droplet diameter *d*_0_. The solid red circle represents the analytical result. The empty circle represents the experimental data. The error bar is twice of standard deviation.

**Figure 6:**
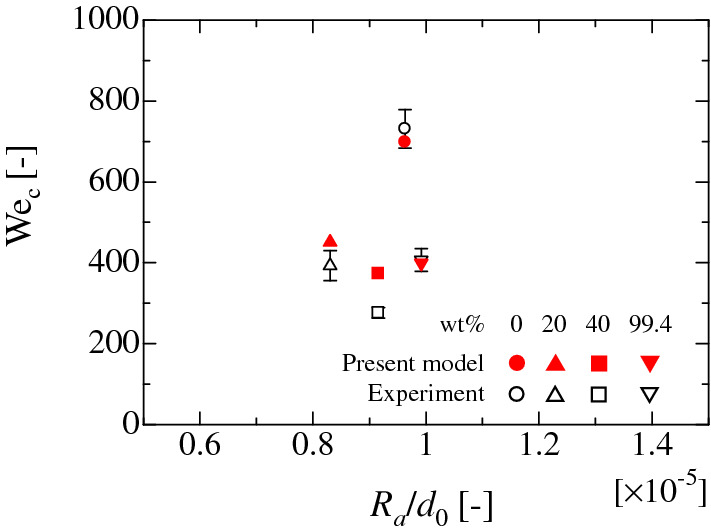
Comparison of the critical We calculated by Eqs. (A10) and (A18) with the experimental data for water–ethanol binary-mixture droplets on the solid substrates of SR. The horizontal axis reflects the arithmetical mean-roughness value *R*_a_ normalised by the initial droplet diameter *d*_0_. The solid red circle represents the analytical result. The empty circle represents the experimental data. The error bar is twice of standard deviation.

Figure 5 shows the comparison of the critical We with the calculated values by the present model for each liquid on PC substrates. The present splashing model shows good agreement with the experimental values in each case. The spreading contact-area diameters at the first stationary condition are also effectively predicted by the modified energy balance equation, as shown in Figs. S3–S6. Figure 5a shows that the critical We becomes small as the relative roughness *R*_a_/*d*_0_ increases. This tendency is related to the static wetting condition^36^. As the surface roughness increases, the hydrophobicity of water droplet (0 wt.%) also increases, corresponding to the Cassie situation in which an air pocket exists between the liquid and solid. This tendency also appears in the spreading behaviour at the stationary condition (Fig. S3). Therefore, the incomplete wetting situation would induce a morphological effect on the circumferential instability of the liquid film and results in the low critical We for splashing. Considered the static wetting condition, the case for (b) 5 wt.% can be also considered to be following the same mechanism. Conversely, for moderate ethanol concentrations of (d) 40 wt.% and (e) 70 wt.%, the critical We increases as the relative roughness increases. In these cases, the wettability of the static droplet becomes large as the surface roughness increases^36^. Therefore, the increase in the adhesion to the solid substrates results in the increase of the critical We for the splashing. The case for (c) 20 wt.% would be a transition region from (a) to (d). In the case of (f) 99.4 wt.%, the contact angle of the static droplet is almost 0 [deg.] on the smooth and rough PC substrates^36^. Therefore, the adhesiveness to each solid substrate is mostly similar, which would result in the similar critical We in each solid substrate. The cases for pure water, 20 wt.%, 40 wt.% and 99.4 wt.% ethanol on the SR substrate are shown in Fig. 6. From the results, the calculated values are in good agreement with the experimental data for hydrophobic case. Here, for the SR substrate with weak adsorption effect on the wettability, the Eq. (12) (or Eq. A10) without the additional terms can be solved if there is no microstructure on the substrate^26,27^. These results prove the validity of the present model.

**Figure 7:**
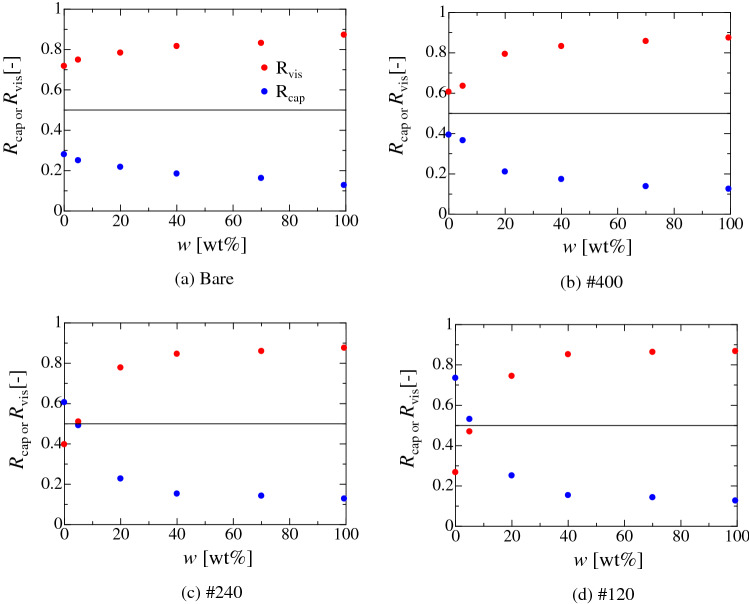
Liquid mass-concentration dependency on the non-dimensional energies of capillary (*R*_cap_) and viscous dissipation (*R*_vis_) for each solid substrate under the splashing condition. *R*_cap_ and *R*_vis_ are evaluated by (*E*_sprd_ + *E*_def_ + *E*_additional_)/(*E*_kine_ + *E*_surf_ + *E*_grav_) and *E*_vis_/(*E*_kine_ + *E*_surf_ + *E*_grav_), respectively. Additional term *E*_additional_ that is related to the adsorption energy (*E*_ads_) and the infiltration work (*E*_infil_) is included in *R*_cap_. The red and blue circles represent *R*_vis_ and *R*_cap_, respectively. The black solid line represents *R*_cap_ = *R*_vis_. *R*_cap_ and *R*_vis_ hold the relation of *R*_cap_ + *R*_vis_ = 1.

Figure 7 shows the liquid concentration dependence on the ratio of non-dimensional energies for the capillary (*R*_cap_) and the viscous dissipation (*R*_vis_) for each solid substrate under the splashing condition. *R*_cap_ and *R*_vis_ are *E*_cap_/*E*_ini_ and *E*_vis_/*E*_ini_, respectively. Here, *E*_cap_ = *E*_sprd_ + *E*_def_ + *E*_additonal_ and *E*_ini_ = *E*_kine_ + *E*_surf_ + *E*_grav_^27^. For the bare substrate (Fig. 7a), the splashing occurs in the viscous dissipation dominated region for all liquid concentrations. However, in the low-ethanol concentration region, *R*_cap_ gradually increases with the surface roughness and splashing occurs in the capillary dominated region for the #120 substrate for water and 5 wt.% ethanol. These results indicate that the critical We is small for the splashing in the capillary region. Conversely, the effect of wettability on the splashing behaviour becomes small as the hydrophilicity increases (high-ethanol concentration region). The difference in the critical We becomes small as the ethanol concentration increases (Fig. 5). Similar behaviour would be observed for metal substrates because the surface free energy of the high-surface energy solid is larger than that of the low-surface energy solid, such as polymer or plastic substrate. In the present study, the maximum and minimum values of the critical capillary numbers (Ca) where the splashing occurs are 0.23 and 0.03, respectively. Therefore, the effect of wettability on the splashing behaviour may disappear if the critical Ca exceeds 0.23 in the present study^56^. Notably, this tendency may depend on the combination of the solid surface morphology/wettability and liquid property/composition.

## Conclusion

In the present study, the droplet splashing behaviours for water–ethanol binary-mixture liquids to some low-surface-energy solids were experimentally investigated. Then, the theoretical model for the prediction of the splashing conditions was developed, considering the pressure balance for the liquid film. In addition, the energy balance equation was modified for both smooth and rough solid surfaces. The modified energy balance equation can predict the spreading factor on the rough solid surface in the deposition region. Furthermore, analytical results obtained by solving the pressure balance equation combined with the modified energy balance equation are in good agreement with the experimental data for the critical Weber number for splashing. The result indicates that the splashing conditions depend on the wettability between the solid and liquid, and the solid surface roughness in addition to the viscosity. In particular, the effect of adsorption on the wettability is an important factor. The present model mainly focuses on the processed surface that is not so rugged, and relatively smooth surface (processed by sandpaper), which corresponds to We_ε_ $\le$ 1 based on the classification by Garcia-Geijo et al.^20^. However, prior to our study, no theoretical model could predict both the splashing conditions for the droplets and the spreading contact-area diameter at the first stationary condition, such as the maximum spreading diameter on the smooth and rough solid surfaces. Although the present model cannot apply to the rugged solid surface where the contact angle is hard to define, predicting the splashing condition on such rugged solid surfaces could be useful as the basic model or methodology.

## Supplementary Information

Supplementary Information.
