# Supplementary material for: Predicting the splash of a droplet impinging on solid substrates
Source: Sci Rep. 2022 Mar 24;12:5093. doi: 10.1038/s41598-022-08852-3 (PMC8948286; doi:10.1038/s41598-022-08852-3)
Supplement: Supplementary file 1 — Supplementary Information. [file 41598_2022_8852_MOESM1_ESM.docx]

Predicting the splash of a droplet impinging on solid substrates

Yukihiro Yonemoto^1,*^, Kanta Tashiro^2^, Kazuki Shimizu^2^, Tomoaki Kunugi^3,**^

^1^ Division of Industrial Fundamentals, Faculty of Advanced Science and Technology, Kumamoto University, 2-39-1, Kurokami, Chuo-ku, Kumamoto-shi, Kumamoto, 860-8555, Japan

^2^ Department of Mechanical and Mathematical Engineering, Kumamoto University, 2-39-1, Kurokami, Chuo-ku, Kumamoto-shi, Kumamoto, 860-8555, Japan

^3^ College of Energy Engineering, Zhejiang University, 38 Zheda Road, Hangzhou, Zhejiang Province, 310027, People’s Republic of China * Corresponding author: yonemoto@mech.kumamoto-u.ac.jp ** Second corresponding author: kunugi.tomoaki.85s@st.kyoto-u.ac.jp

**S1. Laplace pressure exerted on the tip of the liquid film (Equation (3))**

Figure 4 shows a model of the simplified liquid film. Laplace pressure exerted on the tip of the liquid film is modelled based on the Young–Laplace equation, as shown in Fig. 4. The Laplace pressure is modelled by considering the surface tension *f*_tip_ exerted on the arc lengths characterised by *r*_0_ and *r*_film_ and the effective area *A*_eff_ of the liquid film influenced by *f*_tip_.

The surface tension *f*_tip_ mainly comprises two components: *f*_tip1_ and *f*_tip2_. *f*_tip1_ is characterised by *r*_film_, as shown in Fig. 4 (b). The component *f*_tip1_ would exert on the arc length 2π*r*_film_/2. Conversely, the component *f*_tip2_ is characterised by *r*_0_ (= *d*_0_/2), as shown in Fig. 4 (a). The concept of the component *f*_tip2_ is the same as that of *f*_tip1_. However, the liquid film could eject the secondary droplets because of the circumferential instability as in the finger shape and not the disc-like shape. We assume that this instability wave can be simply modelled by the liquid film, as shown in Fig. 4 (c). The concave and convex natures of the liquid film in the circumferential direction are simplified by the projection concept, such as a circle whose circumferential length is characterised by π*d*_0_. If the i-th wavelength comprising concave and convex parts is represented by *L*^(i)^_conc_ and *L*^(i)^_conv_, respectively, the total arc length can be approximated by $\sum L_{conc}^{(i)}+\sum L_{conv}^{(i)}\approx2\pi r_{0}$ that leads to $2\sum L\approx2\pi r_{0}$ by considering the assumptions for the average lengths of $\sum L_{conc}^{(i)}=\sum\bar{L}_{conc}^{(i)}$ and $\sum L_{conv}^{(i)}=\sum\bar{L}_{conv}^{(i)}$ with $\bar{L}_{conc}^{(i)}\approx\bar{L}_{conv}^{(i)}=L$. Therefore, the total arc length (red arc length) can be expressed by 2π*r*_0_/2 if a secondary droplet is assumed to eject from every convex position along the circumference. Thus, based on this concept, the surface tension *f*_tip_ can be represented by the following relation:

$f_{tip}=\frac{2\pi r_{film}}{2}\sigma_{\lg}+\frac{2\pi r_{0}}{2}\sigma_{\lg}$. (A1)

In addition, the effective area *A*_eff_ on which the surface tension *f*_tip_ exerts can be also modelled based on the image in Fig. 4 (c) and represented by the following relation because of the assumption for every other ejection of the secondary droplet along the circumference.

$A_{eff}\approx h_{film}\frac{2\pi r_{0}}{2}$. (A2)

From Eqs. (A1) and (A2), the Laplace pressure exerted on the tip of the liquid film is derived as follows:

$\begin{matrix} P_{Lap}\approx\frac{f_{tip}}{A_{eff}} \\ =\left(\frac{1}{d_{0}}+\frac{1}{h_{film}}\right)\sigma_{\lg} \end{matrix}$. (A3)

**S2. Modelling of the wettability effect on the secondary droplet**

The precise prediction of the size of the secondary droplet ejected from the liquid film is very difficult. Therefore, in the present study, the secondary droplet volume is characterised by the thickness of the liquid film *h*_film_ as *V*_2nd_^drop^ = π*h*_film_^3^/6. In addition, the secondary droplet volume *V*_2nd_^drop^ is assumed to be proportional to the droplet volume *V*_stp_ characterised by *h*_stp_. Here, *V*_stp_ is π*h*_stp_^3^/6, and *h*_stp_ is evaluated assuming a disc-like shape of the droplet as *V*_0_/π*r*_stp_^2^, where *r*_stp_ is the spreading contact-area radius at the first stationary condition. Furthermore, from the experimental observation, such as the splashing behaviours on the bare substrate shown in Fig. 3, the thickness of the liquid film and size of the secondary droplet seem to increase as the wettability increases. Thus, the secondary droplet size would be affected by the wettability because the spontaneous capillary-driven spreading will occur when the droplet contacts with the solid substrate [38, 39]. Even for the impact of a solid body into a liquid bath, the wettability affects the splashing behaviour of the liquid [40]. Therefore, in the present study, it is postulated that the ejected droplet volume *V*_2nd_^drop^ is related to the ratio of the work of adhesions *W*_adh_ = σ_lg_(1+cosθ) between the moment of impact $W_{adh}^{\text{θ}=\pi/2}$ and its subsequent spreading $W_{adh}^{\text{θ}=\bar{\text{θ}}^{i}}$, where the contact angle at a moment of impact is assumed to be 90 [deg.]. Here, $\bar{\text{θ}}^{i}$ (*i* = bare or rough) is the average contact angle of $\theta_{st}^{i}$ and $\theta_{stp}^{i}$ defined in Eq. (A12): $\theta_{st}^{i}$ and $\theta_{stp}^{i}$ represent the static contact angle and the contact angle, respectively, at which the contact-area radius reaches the stationary condition on each solid substrate.

$V_{2nd}^{drop}=\frac{\overline{W_{adh}^{\text{θ}}}}{W_{adh}^{\text{θ}=\pi/2}}V_{stp}$. (A4)

In Eq. (A4), $\overline{W_{adh}^{\text{θ}}}$ is defined as follows:

$\overline{W_{adh}^{\text{θ}}}=(1-f(\bar{\text{θ}}^{i}))W_{adh}^{\text{θ}=\pi/2}+f(\bar{\text{θ}}^{i})W_{adh}^{\text{θ}=\bar{\text{θ}}^{i}}$. (A5)

Equation (A5) is the weighted average of $W_{adh}^{\text{θ}=\pi/2}$ and $W_{adh}^{\text{θ}=\bar{\text{θ}}^{i}}$to consider the spreading process from the impact. Here, *f* (θ^spread^) is defined as follows [27]:

$f(\bar{\text{θ}}^{i})=\frac{1+\cos\bar{\text{θ}}^{i}}{2}$. (A6)

Finally, the following relation can be derived as follows:

$h_{film}=\left(1+f(\bar{\text{θ}}^{i})\cos\bar{\text{θ}}^{i}\right)^{1/3}\frac{2}{3}\frac{d_{0}^{3}}{d_{stp}^{2}}$. (A7)

**S3. Additional energy terms in the energy balance equation**

Wettability of droplet on a solid substrate is affected by the surface morphology and interaction between the solid and liquid molecules. In the present study, to consider the effect of surface roughness on the wetting phenomena, the additional energy terms related to the adsorption (*E*_ads_) and infiltration into the surface morphology (*E*_infil_) are added into the conventional energy balance equation. Furthermore, the energy can be estimated from the energy for the surface deformation of droplet *E*_def_ because the adsorption and the surface morphology affect the droplet surface deformation through the contact line motion. The concrete expression for *E*_ads_ is modelled as follows:

$E_{ads}=g_{ads}E_{def}$, (A8)

where *g*_ads_ is defined in Eq. (15). In Eq. (A8), the negative value for *g*_ads_, reflecting the reduction in the energy for the surface deformation corresponds to the relative increase in the other energies in the energy balance equation, leading to the spreading of droplet. The positive value indicates that the energy is consumed in the surface deformation rather than spreading. Figure S1 shows the relationship between *g*_ads_ (= *E*_ads_/*E*_def_) and σ_lg_. From this figure, it is found that the reduction of the adsorption energy to the surface deformation increases as the surface tension becomes small, indicating that the deformation of the liquid surface contributes to the contact line movement (radial spreading) if the wettability is good. As for *E*_infil_, the following relation is assumed:

$E_{infil}=g_{infil}E_{def}$, (A9)

where *g*_infil_ is defined as Eq. (17). Figure S2 shows the relationship between *g*_infil_ (= *E*_infil_ / *E*_def_) and σ_lg_. As shown in this figure, *g*_infil_ increases as the surface tension decreases (increase in the ethanol concentration) and the surface roughness increases. For purified water, *g*_infil_ becomes large as the surface roughness becomes large, but the magnitude of *g*_infil_ is small compared with that of the low surface tension liquid. This tendency is physically related to the Wenzel or Cassie state. For water, the hydrophobicity becomes strong as the surface roughness increases because of the air pockets in the grooves of the surface. Thus, the infiltration area for the low surface tension case is larger than that of the high-surface tension case, such as water, represented by the difference in the magnitudes of *g*_infil_, as shown in Fig. S2.

**S4. Prediction model for the spreading factor of the contact-area diameter on smooth and rough solid substrates**

The parameters A, B, C, and D are determined by the relationship between the spreading contact-area diameter at the first stationary condition (*β*_stp_) and We based on the following modified energy balance equation:

$\frac{We}{3}-\frac{27}{16}\frac{r_{0}^{2}}{h_{stp}^{2}}k^{2}\beta_{stp}\frac{We}{Re}-(1-\cos\bar{\text{θ}}^{i})\beta_{stp}^{2}+\frac{h_{stp}}{r_{0}}\beta_{stp}\sin\bar{\text{θ}}^{i}+\frac{2}{3}\frac{\rho_{l}gr_{0}h_{stp}}{\sigma_{\lg}}+4-\frac{S_{def}}{\pi r_{0}^{2}}-\frac{\left(g_{ads}+g_{infil}\right)S_{def}}{\pi r_{0}^{2}}=0$. (A10)

where the parameters *k* and $\bar{\text{θ}}^{i}$ are defined as follows:

$k=\left(\frac{1+\cos\overline{\text{θ}}^{bare}}{1+\cos\overline{\text{θ}}^{i}}\right)^{1/2}$, (A11)

$\bar{\text{θ}}^{i}=\frac{\text{θ}_{st}^{i}+\text{θ}_{stp}^{i}}{2}$ $(i=bareorrough)$. (A12)

In addition, *S*_def_ is defined as follows [27]:

$S_{def}=f(\bar{\text{θ}}^{i})S_{harm}+(1-f(\bar{\text{θ}}^{i}))S_{disc}$, (A13)

and

$f(\bar{\theta}^{i})=\frac{1+\cos\bar{\text{θ}}^{i}}{2}$, (A14)

$S_{harm}=\frac{2S_{cap}S_{disc}}{S_{cap}+S_{disc}}$, (A15)

$S_{disc}=\pi r_{stp}^{2}+2\pi r_{stp}h_{stp}$, (A16)

$S_{cap}=\pi\left(r_{stp}^{2}+h_{stp}^{2}\right)$. (A17)

In Eq. (A12), the values of θ_st_ and θ_stp_ for PC are shown in Table S1. In the complete wetting cases for 40 wt.% (#240 and #120), 70 wt.% (#400, #240 and #120) and 99.4 wt.% (bare, #400, #240 and #120) [30], the apparent static contact angle θ_st_ is considered as θ_st_$\approx$0. The values of θ_st_ and θ_stp_ for SR are shown in Table S2. Figures S3–S6 show the examples for the results of the relationship between *β*_stp_ and We of four PC solid substrates for the water–ethanol binary-mixture liquids of 0 wt.%, 20 wt.%, 70 wt.% and 99.4 wt.%. In each figure, four solid surface conditions of Bare, #400, #240 and #120 are shown in (a), (b), (c) and (d), respectively. The red solid line is the analytical solution of Eq. (A10) with A = 8.135, B = 7.823, C = −0.3797 and D = 1.792. The black dashed line in (b), (c) and (d) is depicted as a reference line of the result for the bare substrate. The symbols are the experimental results. From these figures, the present model shows good agreement with the experimental data for the spreading contact-area diameter at the first stationary condition on smooth and rough solid substrates. For the SR substrate with weak adsorption effect on the wettability, the equation (A10) or (12) without the additional terms can be solved if there is no microstructure on the substrate [26, 27].

In the present study, the splashing condition is predicted by solving the energy balance equation with *k* parameter and the additional energy terms (Eq. (A10)) and the following pressure balance for the liquid film:

$\left(\frac{3}{2}+\frac{27}{16}k\frac{We}{\mathrm{Re}}\right)\beta_{stp}^{2}=\left(1+f(\bar{\text{θ}}^{i})\cos\bar{\text{θ}}^{i}\right)^{1/3}\left(\frac{9k^{2}}{128}We-1+\frac{\rho_{l}gd_{0}^{2}}{\sigma_{\lg}}\right)$. (A18)

The intersection of Eq. (A10) with Eq. (A18) represents the beginning of the splashing, which means that the intersection point is the boundary separating the deposition and splashing. Figure S7 shows the relationship between *β*_stp_ and We with the conditions for water droplet (4.5 μL) on the bare PC substrate. The black and red solid lines are calculated by Eqs. (A10) and (A18), respectively. Considering the inequality sign in Eq. (10), the model indicates that the splashing occurs in the blue solid line region.

**S5. Evaluation of our experimental data based on some existing models**

To evaluate the validity of our experimental data, two existing models are used as references in the present study. One is proposed by Almohammadi and Amirfazli (AA model) [16], in which the splashing behaviour in a wide range of viscosities and surface tensions is experimentally investigated. Then, the splashing conditions are categorised into two patterns and the following relations are proposed:

Pattern I: $We=K_{1}\mathrm{Re}^{\beta_{1}}+K_{2}\mathrm{Re}^{\beta_{2}}$, (A19)

Pattern II: $We=K_{3}\mathrm{Re}^{\beta_{3}}$. (A20)

In these relations, the values of β_1_, β_2_ and β_3_ are 1, −1/2 and 1, respectively. Then, the parameters *K*_1_, *K*_2_ and *K*_3_ depend on the wettability. In pattern I, *K*_1_, *K*_2_ and *K*_3_ are 0.06, 6762 and 1.6 for a hydrophilic substrate with receding θ_R_ and advancing θ_A_ contact angles > 5 [deg.]. In pattern II, *K*_1_, *K*_2_ and *K*_3_ are 0.05, 6762 and 1.2, respectively, if the surface is very hydrophilic (θ_R_ and θ_A_ < 5 [deg.]) or hydrophobic. Another model is proposed by Gordillo and Riboux (RG model) [20, 28], where the local liquid-film behaviour in splashing is thoughtfully investigated. In the present study, the experimental data corresponds to the condition We_ε_ ≤ 1. Thus, the following relation is used to evaluate the splashing criterion *K*_c_:

$K_{c}=K_{l}\left(\frac{\mu_{g}}{\mu_{l}}\right)OhWe^{5/6}$. (A21)

$K_{l}=\frac{3}{\tan^{2}\alpha}\ln\left[A\left(\frac{\mu_{l}}{\mu_{\mathrm{g}}}\right)^{3/4}Oh^{-1/4}\left(We\frac{\lambda_{g}}{r_{0}}\right)^{-1}\right]$. (A22)

In this model, We is defined using the initial droplet radius *r*_0_ as ρ*u*^2^*r*_0_/σ_lg_, where ρ is liquid density, *u* is the droplet impinging velocity, σ_lg_ is the surface tension between the gas and liquid, λg is the mean free path of the gas molecules, Oh is the Ohnesorge number (=We^1/2^Re^−1^), and μ_g_ and μ_l_ are the gas and liquid viscosities, respectively. In Eq. (A22), A and α are set as 0.011 and 60 [deg.], respectively. Here, *K*_c_0.034 is the splashing condition [20]. Thus, the splashing does not occur if *K*_c_ < 0.034.

Figure S8 shows the comparison of the present experimental data with the AA model using the values of *K*_1_, *K*_2_ and *K*_3_ in pattern II. The present experimental data for the smooth solid substrate (Fig. S8 (a)) are included in the splashing area surrounded by the two empirical equations (A19) and (A20), whereas the experimental data in other cases (Fig. S8 (b) to (d)) are partly included in the area. This is because Eqs. (A19) and (A20) do not cover all the cases for the rough solid surface. However, this result indicates reliability because the experimental data for the smooth solid surface agrees well with the existing model.

Figure S9 shows the results based on the RG model. The vertical and horizontal axes represent the calculated *K*_c_ values and the mass concentration of the liquid *w*, respectively. From this result, it is found that the RG model agrees well with the bare substrate although there are deviations from the threshold of *K*_c_0.034 in other substrates. Here, the average values of *K*_c_ and twice the standard deviation for each solid substrate are 0.034$\pm$0.008 for bare, 0.032$\pm$0.022 for #400, 0.033$\pm$0.024 for #240 and 0.031$\pm$0.027 for #120.

From the above comparisons, our experimental data for the bare substrate agrees well with the two exiting models. Especially, if only the average values of *K*_c_ are considered, the differences in the *K*_c_ values for other roughened substrates from 0.034 are not prominent. This indicates the reliability for the present experimental data for bare and other roughened solid substrates.

Table S1

Table S1 Data for contact angles of θ_st_ and θ_stp_ [deg.] on PC substrates, where θ_stp_ is the contact angle when the contact-area diameter reaches the first stationary condition just after the impingement. Upper and bottom values are θ_st_ and θ_stp_, respectively. The values θ_st_ of the complete wetting cases in 40 wt.% (#240 and #120), 70 wt.% (#400, #240 and #120) and 99.4 wt.% (bare, #400, #240 and #120) are represented by θ_st_$\approx$0 [deg.].

Liquid

0 wt.% (Pure water)

σ_lg_ = 0.0719 Nm^−1^

5 wt.%

σ_lg_ = 0.0563 Nm^−1^

20 wt.%

σ_lg_ = 0.0384 Nm^−1^

40 wt.%

σ_lg_ = 0.0300 Nm^−1^

70 wt.%

σ_lg_ = 0.0256 Nm^−1^

99.4 wt.%

σ_lg_=0.0211 Nm^-1^

#120

#240

Bare

#400

121.6 ± 9.7

140.8 ± 9.3

117.6 ± 4.2

134.6 ± 9.1

110.9 ± 8.3

117.1 ± 4.8

91.8 ± 2.5

113.3 ± 6.2

97.7 ± 6.9

130.3 ± 15.4

100.5 ± 7.9

123.5 ± 13.8

92.1 ± 5.1

113.3 ± 15.5

82.4 ± 2.2

94.0 ± 12.1

53.4 ± 7.0

91.3 ± 24.2

50.2 ± 3.6

86.9 ± 20.8

58.7 ± 4.7

75.6 ± 10.2

64.4 ± 2.7

79.5 ± 8.2

$\approx$0

61.8 ± 13.8

$\approx$0

67.6 ± 15.4

24.0 ± 3.2

75.4 ± 16.0

39.1 ± 1.9

76.7 ± 6.8

26.1 ± 2.9

55.7 ± 9.8

$\approx$0

29.1 ± 8.0

$\approx$0

33.6 ± 11.0

$\approx$0

38.2 ± 11.3

$\approx$0

32.2 ± 8.6

$\approx$0

21.9 ± 7.5

$\approx$0

26.6 ± 4.7

$\approx$0

25.9 ± 6.3

Table S2

Table S2 Data for contact angles of θ_st_ and θ_stp_ [deg.] on SR substrate, where θ_stp_ is the contact angle when the contact-area diameter reaches the first stationary condition just after the impingement. Upper and bottom values are θ_st_ and θ_stp_, respectively.

| Liquid | Bare |
| --- | --- |
| 0 wt.% (Pure water)  σ_lg_ = 0.0719 Nm^−1^ | 112.0 ± 1.9  128.2 ± 9.2 |
| 20 wt.%  σ_lg_ = 0.0384 Nm^−1^ | 82.8 ± 2.6  96.7 ± 14.3 |
| 40 wt.%  σ_lg_ = 0.0300 Nm^−1^ | 68.0 ± 1.6  86.2 ± 13.1 |
| 99.4 wt.%  σ_lg_=0.0211 Nm^-1^ | 36.4 ± 5.1  66.2 ± 5.7 |

Figure S1

σ_lg_ [Jm^-2^]

*E*_ads_ / *E*_def_ [-]

#120

#240

#400

Bare

Figure S1 Relationship between *g*_ads_ (= *E*_ads_/*E*_def_) and σ_lg_. The open circle, solid circle, open triangle and solid triangle represent *g*_ads_ for the bare, #400, #240 and #120 substrates, respectively.

Figure S2

#120

#240

#400

Bare

σ_lg_ [Jm^-2^]

*E*_infil_ / *E*_def_ [-]

Figure S2 Relationship between *g*_infil_ (= *E*_infil_/*E*_def_) and σ_lg_. The open circle, solid circle, open triangle and solid triangle represent *g*_infil_ for the bare, #400, #240 and #120 substrates, respectively.

Figure S3

Bare

Eq. (A10)

*β*_stp_ [-]

*β*_stp_ [-]

(a)Bare

(b)#400

We [-]

We [-]

*β*_stp_ [-]

*β*_stp_ [-]

(c)#240

(d)#120

We [-]

We [-]

Figure S3 Relationship between *β*_stp_ and We for the purified water (0 wt.%) droplet impinging on (a) bare, (b) #400, (c) #240 and (d) #120 PC substrates. In (b), (c) and (d), the black dashed line represents the analytical result for the bare substrate and is depicted as a reference. The error bar represents twice the standard deviation.

Figure S4

(b)#400

(a)Bare

*β*_stp_ [-]

*β*_stp_ [-]

Eq. (A10)

*β*_stp_ [-]

*β*_stp_ [-]

Bare

We [-]

We [-]

We [-]

We [-]

(c)#240

(d)#120

Figure S4 Relationship between *β*_stp_ and We for 20 wt.% droplet impinging on (a) bare, (b) #400, (c) #240 and (d) #120 PC substrates. In (b), (c) and (d), the black dashed line represents the analytical result for the bare substrate and is depicted as a reference. The error bar represents twice the standard deviation.

Figure S5

(a)Bare

(b)#400

*β*_stp_ [-]

*β*_stp_ [-]

*β*_stp_ [-]

Eq. (A10)

*β*_stp_ [-]

Bare

We [-]

We [-]

(c)#240

(d)#120

We [-]

We [-]

Figure S5 Relationship between *β*_stp_ and We for 70 wt% droplet impinging on (a) bare, (b) #400, (c) #240 and (d) #120 PC substrates. In (b), (c) and (d), the black dashed line represents the analytical result for the bare substrate and is depicted as a reference. The error bar represents twice the standard deviation.

Figure S6

(b)#400

(a)Bare

Bare

Eq. (A10)

*β*_stp_ [-]

*β*_stp_ [-]

*β*_stp_ [-]

*β*_stp_ [-]

We [-]

We [-]

(c)#240

(d)#120

We [-]

We [-]

Figure S6 Relationship between *β*_stp_ and We for 99. 4 wt.% droplet impinging on (a) bare, (b) #400, (c) #240 and (d) #120 PC substrates. In (b), (c) and (d), the black dashed line represents the analytical result for the bare substrate and is depicted as a reference. The error bar represents twice the standard deviation.

Figure S7

Splashing

Eq. (A18)

Eq. (A10)

0 wt% (4.5 μL) on Bare

*β*_stp_ [-]

We [-]

Figure S7 Onset of droplet splash for the purified water droplet (0 wt.%) of 4.5 μL. The black and red solid lines are calculated by Eqs. (A10) and (A18), respectively. The intersection point of the two equations is the onset of the droplet splash. Considered Eq. (10), the blue solid line is the splashing region.

Figure S8

(d)#120

(c)#240

(b)#400

(a)Bare

We_c_ [-]

We_c_ [-]

We_c_ [-]

We_c_ [-]

Re_c_ [-]

Re_c_ [-]

Re_c_ [-]

Re_c_ [-]

Figure S8 Comparison of the present experimental data with the existing AA model [16]. Solid lines are evaluated by Eqs. (A19) and (A20). The open circle, solid circle, open triangle, solid triangle, open square and solid square represent the experimental data for the liquids of 0 wt.%, 5 wt.%, 20 wt.%, 40 wt.%, 70 wt.% and 99.4 wt.%, respectively. According to the AA model developed for the smooth solid surface, the area surrounded by the solid lines indicates the splashing condition region.

Figure S9

*w* [wt%]

*K_c_* [-]

Figure S9 Splashing threshold value *K*_c_ for each solid substrate. The circle, filled circle, triangle and filled triangle represent the calculated *K*_c_ values for the bare, #400, #240 and #120 substrates, respectively. According to the RG model, *K*_c_0.034 indicated by the red solid line is the splashing condition [20]. The average values of *K*_c_ and two-standard deviation for each solid substrate are 0.034$\pm$0.008 for bare, 0.032$\pm$0.022 for #400, 0.033$\pm$0.024 for #240 and 0.031$\pm$0.027 for #120, respectively.
